# Monitoring of Early Changes of Circulating Tumor DNA in the Plasma of Rectal Cancer Patients Receiving Neoadjuvant Concomitant Chemoradiotherapy: Evaluation for Prognosis and Prediction of Therapeutic Response

**DOI:** 10.3389/fonc.2020.01028

**Published:** 2020-07-24

**Authors:** Filip Pazdirek, Marek Minarik, Lucie Benesova, Tereza Halkova, Barbora Belsanova, Milan Macek, Lubomír Stepanek, Jiri Hoch

**Affiliations:** ^1^Department of Surgery, 2nd Faculty of Medicine, Charles University and Motol University Hospital, Prague, Czechia; ^2^Elphogene, Prague, Czechia; ^3^Center for Applied Genomics of Solid Tumors (CEGES), Genomac Research Institute, Prague, Czechia; ^4^Department of Biology and Medical Genetics, 2nd Faculty of Medicine, Charles University and Motol University Hospital, Prague, Czechia; ^5^Institute of Biophysics and Informatics, 1st Faculty of Medicine, Charles University, Prague, Czechia

**Keywords:** rectal cancer, neoadjuvant chemoradiotherapy, circulating tumor ctDNA, prediction, prognosis, response, biomarker

## Abstract

**Introduction:** Patients with locally advanced rectal cancer (LARC) are undergoing neoadjuvant chemoradiotherapy (NCRT) prior to surgery. Although in some patients the NCRT is known to prevent local recurrence, it is also accompanied by side effects. Accordingly, there is an unmet need to identify predictive markers allowing to identify non-responders to avoid its adverse effects. We monitored circulating tumor DNA (ctDNA) as a potential liquid biopsy-based biomarker. We have investigated ctDNA changes plasma during the early days of NCRT and its relationship to the overall therapy outcome.

**Methods and Patients:** The studied cohort included 36 LARC patients (stage II or III) undergoing NCRT with subsequent surgical treatment. We have detected somatic mutations in tissue biopsies taken during endoscopic examination prior to the therapy. CtDNA was extracted from patient plasma samples prior to therapy and at the end of the first week. In order to optimize the analytical costs of liquid-biopsy testing, we have utilized a two-level approach in which first a low-cost detection method of denaturing capillary electrophoresis was used followed by examination of initially negative samples by a high-sensitivity BEAMING assay. The ctDNA was related to clinical parameters including tumor regression grade (TRG) and TNM tumor staging.

**Results:** We have detected a somatic mutation in 33 out of 36 patients (91.7%). Seven patients (7/33, 21.2%) had ctDNA present prior to therapy. The ctDNA positivity before treatment reduced post-operative disease-free survival and overall survival by an average of 1.47 and 1.41 years, respectively (*p* = 0.015, and *p* = 0.010). In all patients, ctDNA was strongly reduced or completely eliminated from plasma by the end of the first week of NCRT, with no correlation to any of the parameters analyzed.

**Conclusions:** The baseline ctDNA presence represented a statistically significant negative prognostic biomarker for the overall patient survival. As ctDNA was reduced indiscriminately from circulation of all patients, dynamics during the first week of NCRT is not suited for predicting the outcome of LARC. However, the general effect of rapid ctDNA disappearance apparently occurring during the initial days of NCRT is noteworthy and should further be studied.

## Introduction

Current treatment of rectal cancer is based on a multimodal approach involving surgery, radiation, and systemic therapies (1). The actual therapeutic decision process is based on a precise disease staging with early tumors being preferentially considered for direct surgical treatment. Patients with locally advanced rectal cancer (LARC), that is, stages II and III, should undergo radiation in combination with systemic chemotherapy prior to surgery. This neoadjuvant chemoradiotherapy (NCRT) typically includes 5-fluoropyrimidine administered within either a “long regimen” involving a total dosage 50.4 Gy (45 Gy split into 25 fractions received within 5 weeks and initial boost 5.4 Gy) or a “short regimen” of 25 Gy in five fractions during the first week. Although records show that NCRT in general does not improve the overall survival (OS), it reduces the local recurrence of RC to under 10% of all cases (2). Patients showing appropriate response to NCRT have a significantly better prognosis in the long-term perspective (3, 4).

The clinical and laboratory evaluation following NCRT includes endoscopic examination, MRI, and histopathology (5). The objective response to NCRT may include (i) a complete elimination of the tumor, (ii) partial regression of the tumor size, (iii) elimination or reduction of the number of tumor-positive lymph nodes in the mesorectum, or (iv) any combination thereof. Although a complete clinical response is found in 15–30% of all cases and partial response is reached in 20–5% of all patients, half of the tumors will remain principally unaffected following NCRT (3, 4). At the same time, however, it is known that NCRT induces a variety of adverse side effects, most importantly development of fibrosis due to the post-radiative pelvic damage leading to impairment of the anal sphincter, leading to incontinence and erectile dysfunction. It is also associated with post-operative complications and worsening of post-operative functional results (6, 7). In order to avoid and/or minimize the adverse effects of NCRT, it is highly desirable to modify or even eliminate preoperative NCRT in potential non-responders.

Identification of patients non-responding to NCRT has recently become a focus of sustained clinical research. A wide spectrum of approaches has been investigated including application of various predictive markers. The traditional clinical parameters such as tumor size and distance from anal margin (evaluated by endoscopy and MRI) were complemented by laboratory biomarkers comprising, for example, hemoglobin, carcinoembryonic antigen (CEA) serum levels, or tumor-infiltrating lymphocytes (8). Furthermore, use of a contrast MRI with diffusion-weighted imaging (DWI), which accounts for perfusion and cellular density and thus reflects tumor biology, has also been utilized. However, to this point, study results have been generally contradictory (9–15).

More recently, a role of molecular genetic markers of NCRT response prediction was investigated. Among others, variants of the *KRAS* gene (MIM# 190070), which is mutated in 30–60% sporadic colorectal cancer (CRC), are the most frequently studied genetic “biomarkers” (16). Activating pathogenic variants (henceforward termed in legacy nomenclature “mutations”) in *KRAS* are associated with poor response to biological therapy as monitored by monoclonal anti-epidermal growth factor receptor (anti-EGFR) antibodies (17). Several studies have assessed *KRAS* as a predictive marker for the therapeutic effect of NCRT, indicating better response of the wild type over its mutated alleles (18, 19). This concept, however, has not been confirmed by others (20, 21). In addition, because mutations in the *TP53* gene (MIM# 191170) have also been frequently observed in CRC, it was suggested that it could serve as a potential predictor of resistance to NCRT. Several groups implied that *TP53* gene wild-type constitution and a lower expression of the p53 protein product are both associated with proper therapeutic response (22–24). Nonetheless, these studies have not been universally accepted, and further studies are needed (25, 26).

Detection of DNA circulating in the blood of patients [cell-free DNA (cfDNA)] has recently gained considerable interest as a potentially new class of molecular markers in the area of cancer diagnostics and management (27). CfDNA consists of short DNA fragments released from decomposing tumor cells mainly through necrotic and apoptotic processes as well as active endosomal release. Its levels are significantly elevated in organisms undergoing cellular decomposition induced by immune response such as owing to infection or inflammation as well as cancer. The typical levels of cfDNA range approximately from 10 to 100 ng/ml of plasma (~3,000–30,000 DNA copies/ml) in healthy individuals up to mg/ml levels in cancer patients (28). The part originating from cancerous cells is referred to as a circulating tumor DNA (ctDNA). CtDNA, which represents only a very small fraction of the overall cfDNA, typically contains somatic mutations, which are only present in tumor and not in the “healthy” cells, hence, presents an alternative source for predictive cancer-specific mutation detection. This fact has been intensely investigated giving rise to a whole new area of ctDNA “liquid biopsy-based” diagnostic strategies. Aside from being used as an alternative source of material for tumor diagnostics, the relative changes in ctDNA levels are known to correlate with the overall tumor burden in a given patient, thus indirectly reflecting the overall size and number of cancerous lesions present in the body (29). Therefore, ctDNA evaluation has a great potential as a marker for monitoring the disease and course of the treatment (30). It has long been recognized that in a positive response to chemotherapy, the ctDNA is reduced or eliminated completely from the peripheral circulation (31–33).

According to recent reports, the natural kinetics of cfDNA levels in blood is a bimodal process in which initially high levels are rapidly decreasing within minutes as the cfDNA fragments are being distributed across highly vascularized organs/tissues and in other biological fluids followed by a period of slower elimination processes (34). The elimination process is through DNA degradation by ribonucleases present in the blood with a typical half-life of 1.5–3 h. The equilibrium between release and elimination supports the use of cfDNA as a marker of concurrent processes taking place in the body. It is universally accepted that tumors responding to radiotherapy exhibit cell damage leading to necrosis by which DNA is rapidly released into the bloodstream. Hence, upon administration of the radiotherapy, ctDNA levels should rise momentarily before being removed from the circulation by natural homeostatic processes.

In this study, we investigated tumor-derived DNA in plasma (ctDNA) in a pilot cohort of patients with LARC undergoing NCRT. The aim of the present work was to observe changes in ctDNA levels in rectal cancer patients during the initial days of the NCRT treatment and to correlate these to the overall clinical outcome of RC therapy.

## Patients and Methods

### Patient Cohort and Neoadjuvant Chemoradiotherapy Therapy

Our prospective study included 36 patients with LARC who had been recruited between 2013 and 2017. The group included 27 men and 9 women with an average age of 64.1 years, capable of undergoing repeated blood sampling during therapy. Patient characteristics are listed in Table 1. The study protocol was approved by the ethics committee of Motol University Hospital, and patients confirmed their study participation by signing an informed consent form. Upon initial recommendation of the committee, only patients with good performance status and compliance were included in the study.

**Table 1:** Patient characteristics.

				**TNM staging I**			**TNM staging II**						
**Patient**	**Gender**	**Age**	**Stage**	**T**	**N**	**M**	**T**	**N**	**M**	**L**	**TRG**	**Mutation**	**Baseline ctDNA**
1	M	68	2	3	0	0	3	0	0	3	2	*TP53*	0
2	M	63	2	3	0	0	0	0	0	1	4	*KRAS*	0
3	M	73	3	3	1	0	3	2a	0	2	2	*KRAS+APC*	0
4	M	74	2	3	0	0	3	2a	0	2	1	*KRAS*	0
5	F	73	3	3	1	0	2	0	0	2	2	*PIK3CA*	0
6	M	64	3	4	1	0	3	1c	0	3	1	*APC*	0
7	M	62	3	3	1	0	3	2b	0	3	2	*TP53+APC*	0
8	F	36	3	3	1	0	2	0	0	2	2	*APC*	0
9	F	61	3	3	1	0	0	0	0	2	4	*TP53*	0
10	M	70	3	3	1	0	2	0	0	2	2	*KRAS+PIK3CA*	0
11	M	64	2	3	0	0	2	0	0	2	3	0	0
12	M	79	2	3	0	0	3	1a	0	2	2	*TP53*	0
13	F	59	2	3	0	0	3	0	0	2	1	*KRAS+TP53*	0
14	M	61	3	3	1	0	3	2a	0	2	1	*KRAS+TP53*	x
15	M	62	2	3	0	0	3	0	0	3	2	*TP53/PIK3CA*	0
16	M	53	3	3	1	0	3	1a	0	2	2	*APC*	0
17	M	79	2	3	0	0	2	0	0	1	2	*KRAS*	0
18	M	64	2	3	1	0	3	1a	0	2	2	*TP53*	0
19	M	72	3	2	1	0	2	2b	0	3	2	*BRAF/TP53*	0
20	F	30	3	1	1	0	1	1b	0	2	1	*KRAS/TP53*	x
21	F	64	3	2	1	0	1	0	0	2	2	*KRAS/APC*	0
22	M	60	3	3	1	0	3	0	0	2	2	0	0
23	F	74	3	3	1	0	3	2b	0	2	1	*KRAS*	0
24	M	63	3	3	1	0	1	0	0	2	2	*TP53*	0
25	M	74	3	3	1	0	1	0	0	1	2	*KRAS*	x
26	F	66	2	4	0	0	2	0	0	3	1	*KRAS*	x
27	M	52	3	3	1	0	2	0	0	2	1	*KRAS/APC*	x
28	M	64	2	3	0	0	2	1c	0	2	3	*KRAS*	x
29	M	83	3	3	1	0	3	2a	0	1	2	*KRAS*	x
30	M	74	3	3	2	0	3	0	0	2	1	*KRAS*	0
31	M	62	3	3	2	0	3	2	0	2	1	*TP53*	0
32	F	63	3	3	1	0	0	0	0	3	4	*APC*	0
33	M	58	3	3	1	0	2	0	0	3	2	*KRAS*	0
34	M	58	3	3	0	0	3	0	0	3	2	*KRAS*	0
35	M	63	3	3	1	0	2	1	0	1	1	*KRAS*	0
36	M	62	3	3	1	0	3	0	0	3	2	0	0

*ctDNA, circulating tumor DNA; Stage, clinical stage of the disease; TNM staging I, before treatment; TNM staging II, after surgery according to histopathology report; L, tumor location (1, upper rectum; 2, middle rectum; 3, lower rectum); TRG, tumor regression grade according to Dworak (0, no response; 1, minimal response; 2, moderate response; 3, near complete response; 4, complete response)*.

Initial endoscopic biopsy was performed, and the tumor was histologically verified. Staging of the disease was determined based on the CT and MRI (Table 1), and the respective treatment was protocol based on these examinations. Tumor tissues (typically a total of three samples acquired by biopsy forceps) collected during the initial endoscopy prior to oncological treatment were immediately post-operatively frozen at −30°C and sent to the collaborating laboratory for genetic testing. Subsequently, tumor tissues were examined for the presence of the most common mutations previously observed in CRC (comprising *KRAS /MIM# 190070/, TP53 /MIM# 191170/, APC /MIM# 611731/, PIK3CA /MIM#* 171834/, *BRAF /MIM# 164757/*, and *CTNNB1 /MIM# 166806/*). Plasma was obtained by centrifugation from blood samples taken prior to and during NCRT.

All patients underwent NCRT consisting of 50.4 Gy of radiation and concomitant administration of Xeloda™ (*capecitabine*) at a dose of 825 mg/m^2^. Irradiation was carried out by 25 fractions with initial boost of 5.4 Gy. At the end of the first week of NCRT, another blood sample was taken for ctDNA examination. A control MRI of the pelvis was performed 6 weeks after termination of NCRT. At 8–10 weeks after the end of NCRT, all patients with LARC underwent surgery. Biopsy samples were evaluated in detail by an expert histopathologist using standard TNM staging. In addition, the “Dworak histopathological tumor regression grade” (TRG) was also determined. Patients were followed up for at least 3 years after surgery. Standard tumor marker examination, colonoscopy, and computed tomography (CT) imaging were performed at regular intervals.

### Tissue and Circulating Tumor DNA Mutation Testing

DNA extraction from tumor tissue bioptates and plasma was performed using standard spin-column procedures. A GenElute™ Mammalian Genomic DNA Miniprep Kit (Sigma Aldrich, St. Louis, Missouri, USA) was used for extraction from the tissue samples. Extraction of ctDNA from blood plasma samples was performed using NucleoSpin Plasma XS kit (Macherey-Nagel, Düren, Germany), the volume of plasma processed was 600 μl, yielding typically between 5 and 50 ng of cfDNA per sample determined by Qubit 2.0 Fluorometer (Life Technologies, Camarillo, CA).

**Figure 1:**
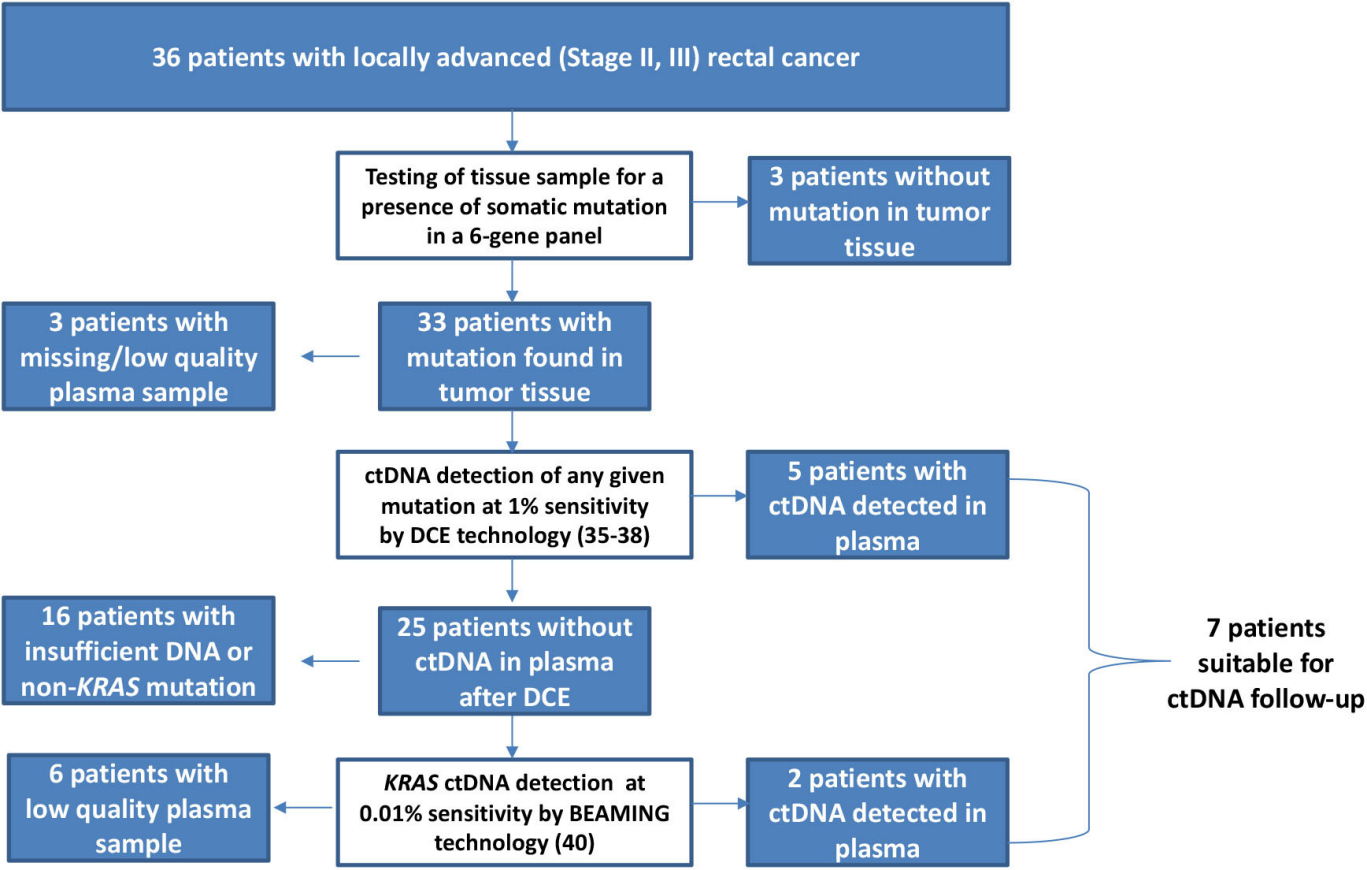
A multilevel cascade testing algorithm for evaluation of circulating tumor DNA (ctDNA) involving standard [denaturing capillary electrophoresis (DCE)] and high-sensitivity (BEAMING*) approaches. *The BEAMING technology was performed using an experimental setting on a set of archived ctDNA samples. This research setting is different from that of protocols applied in regular testing under the IVD-CE certification.

Similarly to works of others (31–33), the mutation analysis of tissue samples was focused on a panel of selected oncogenes, with the highest proportion of somatic mutations in rectal cancer according to the international “COSMIC database” (https://cancer.sanger.ac.uk/cosmic). This panel included the hotspots in *KRAS, BRAF, PIK3CA*, and *CTNNB1*, as well as selected areas of tumor suppressor genes *APC* (mutation cluster region) and *TP53* (exons 5–8). Mutation analyses were performed using the denaturing capillary electrophoresis (DCE) method using the previously described experimental parameters (35–38). Somatic mutations detected in the tissue samples were subsequently examined in samples of cfDNA obtained from plasma. Genetic testing was conducted using two separate methods as discussed further. A subgroup of ctDNA samples assigned as negative by DCE method (39) was subsequently retested using a high-resolution “BEAMING assay” (40) directed at the detection of *KRAS*-specific ctDNA and provided by an external contracted laboratory (Department of Pathology, Jessenius Medical Faculty of Comenius University in Martin, Slovakia). The details of the multilevel ctDNA testing approach are illustrated in Figure 1 and further detailed in the *Discussion* section.

### Statistical Methods

All statistical analyses were performed using R language for statistical computing and graphics (41). Associations between survival period and other predictors such as the presence of ctDNA prior and during NCRT were analyzed using the Cox proportional hazard model and *t*-tests and plotted using boxplots and Kaplan–Meier curves, respectively. Outputs with *p*-values below 0.05 were assumed to be statistically significant.

## Results

**Figure 2:**
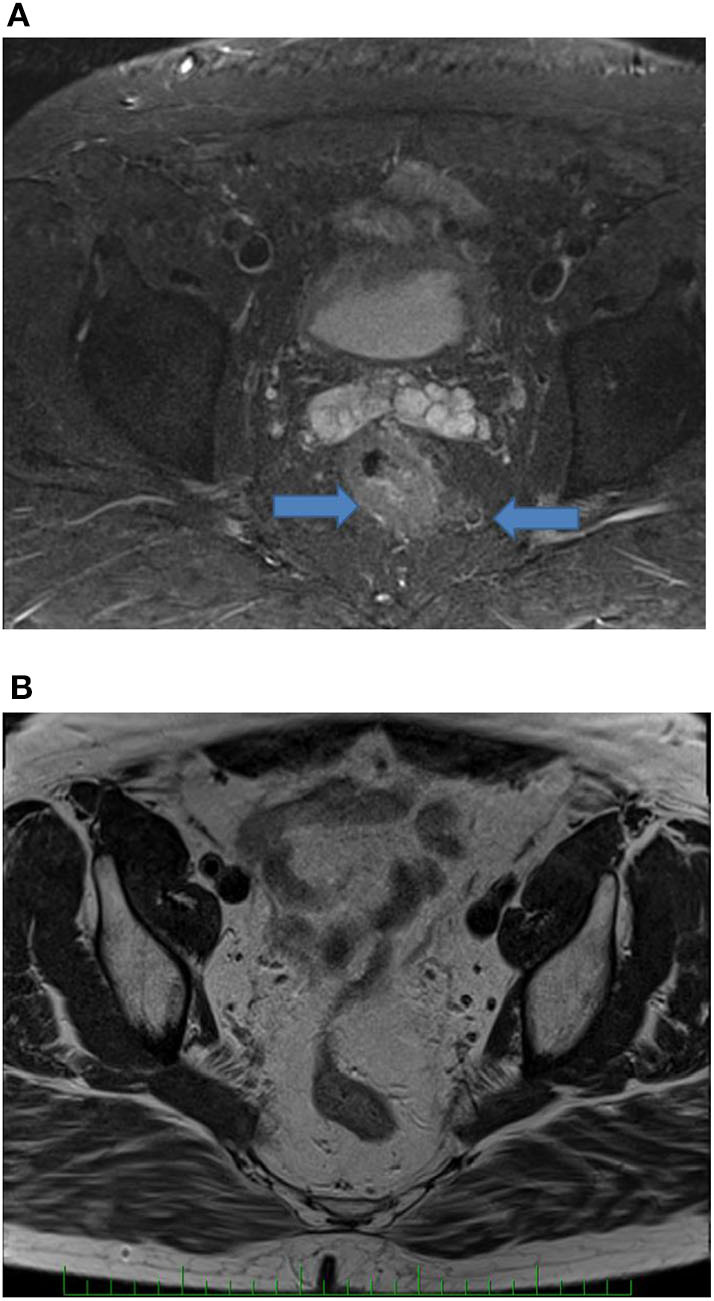
MRI of the rectal tumor (initially T3, N1) before **(A)** and after **(B)** treatment. Arrows show tumor and enlarged lymph node. After treatment, no tumor and lymph nodes are presented (complete clinical tumor response).

**Figure 3:**
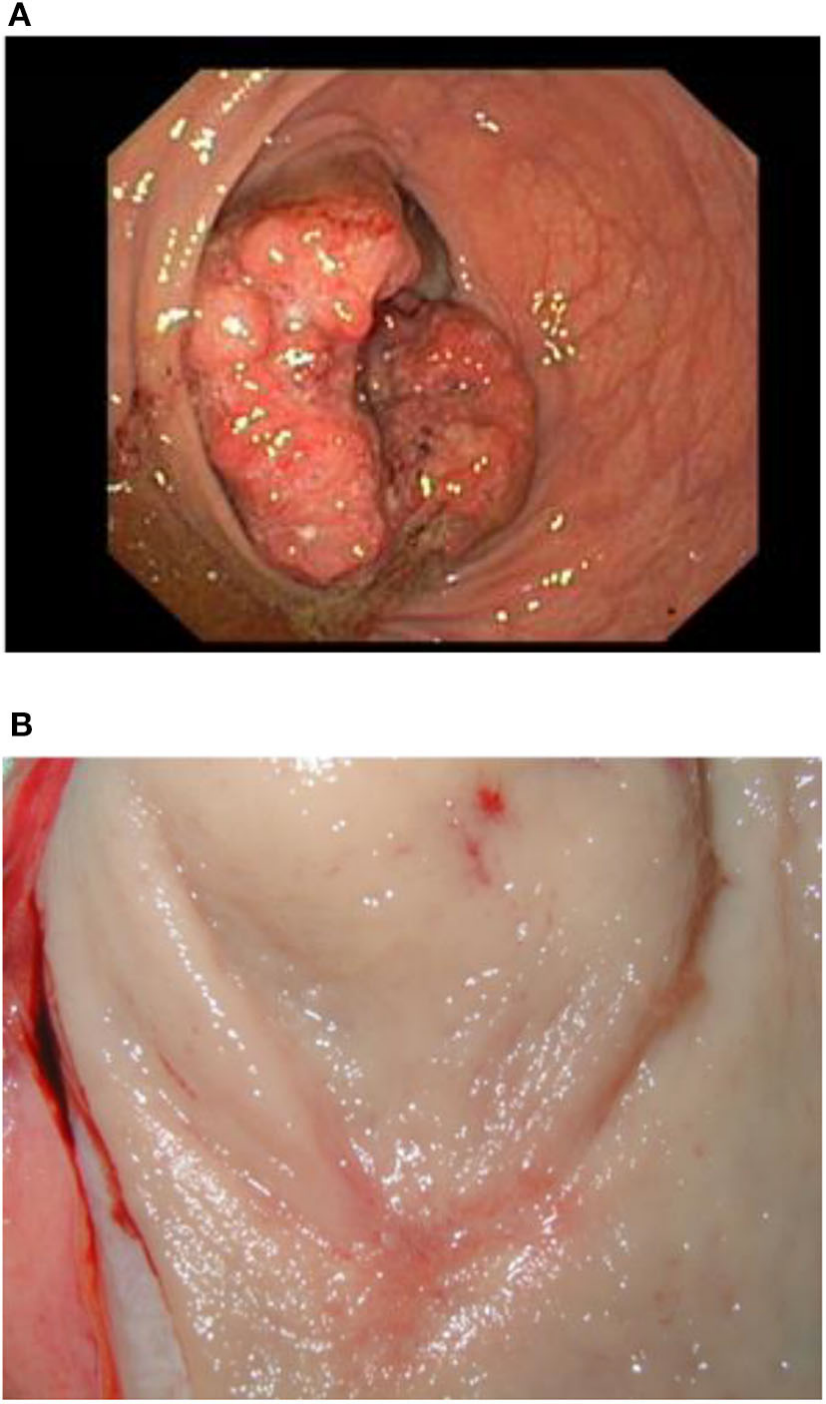
Illustration of a complete therapy response in a patient. Pretreatment endoscopic examination of the tumor **(A)** and surgical specimen **(B)**.

The objective response to NCRT is included in patient characteristics listed in Table 1. The best response characterized by Dworak TRG score of 3 or 4 was observed in five patients (5/36, 13.9%), whereas 11 patients (11/36, 30.5%) showed none or very limited tumor regression (TRG score 0 or 1). Both results were in a range of typically observed response frequencies as described by others (3, 4). When evaluating the effect of NCRT on the disease stage, some patients exhibited a shift toward the lower TNM (such as that illustrated in Figures 2, 3) mainly owing to the reduction of tumor mass and nodal status.

As the tumor tissue biopsies were evaluated prior to therapy, somatic mutations were detected in samples from 33 out of 36 patients (91.7%) using the six-gene panel. As expected, the mutation testing has revealed the presence of combinations of multiple mutations, mainly with concurrent presence of *KRAS* or *TP53* with another mutation type (shown in Table 1). There was no relation between the presence of a specific mutation (or a mutation combination) in the tumor tissue and the ultimate outcome of NCRT evaluated by either TRG or TNM staging.

**Figure 4:**
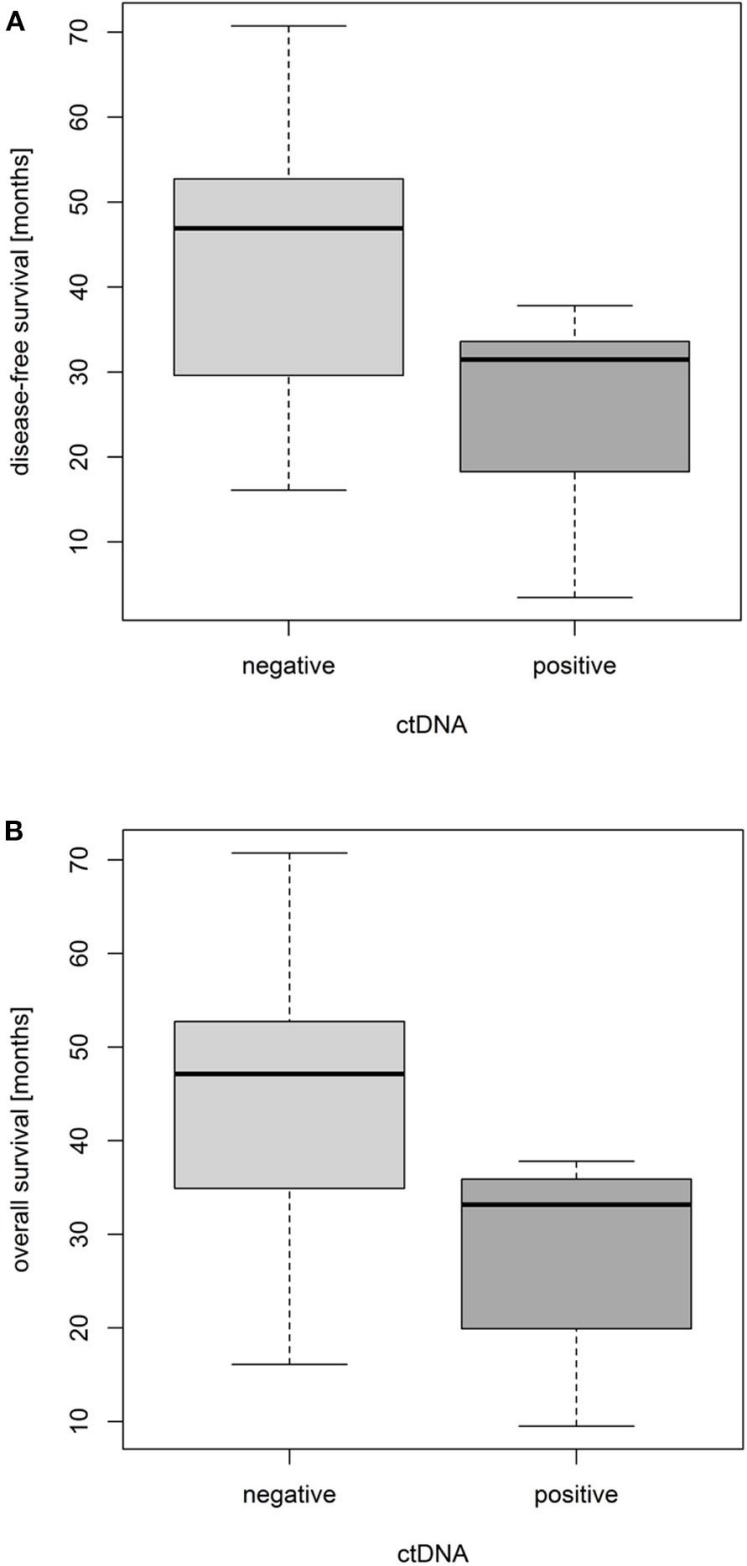
Impact of pretreatment circulating tumor DNA (ctDNA) positivity on disease-free survival **(A)** and overall survival **(B)** of locally advanced rectal cancer (LARC) patients.

**Figure 5:**
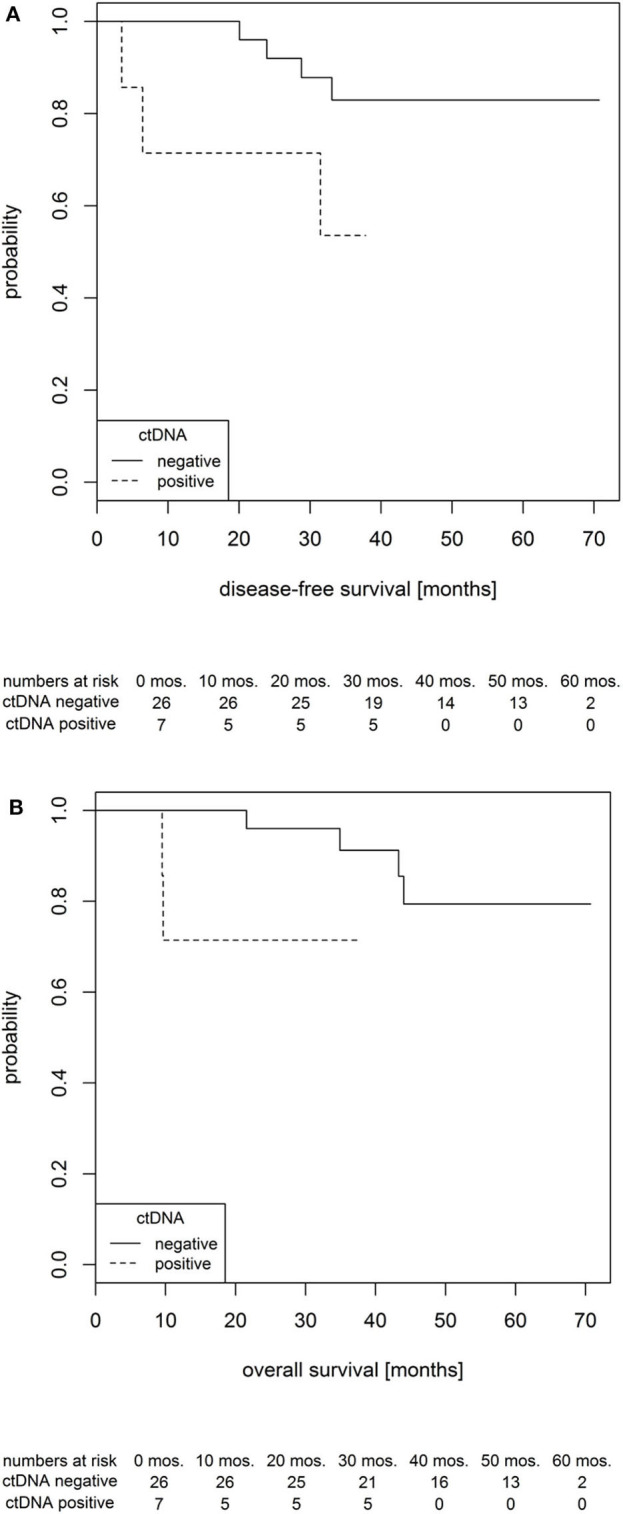
Probability of disease-free survival **(A)** and overall survival **(B)** in locally advanced rectal cancer (LARC) patients according to circulating tumor DNA (ctDNA) status prior to the therapy onset.

With the combination of the low-resolution and high-resolution methods, ctDNA was detected in plasma samples of seven patients prior to NCRT (7/33, 21.2%), and it showed a prognostic role. Whereas, the overall probability of a 3-year survival in all patients was 86.7%, the value was 91.2% in ctDNA-negative subgroup and 71.4% in ctDNA-positive subgroup. Hence, as shown using boxplots in Figure 4, comparing both groups of patients with positive and negative ctDNA and not considering the time-event dimension and proved by *t*-tests, the ctDNA-positive status prior to NCRT was significantly associated with an shorter disease-free survival (DFS) and a shorter OS by an average of 1.47 and 1.41 years, respectively [*t*_(DFS)_= 2.95, *df*_(DFS)_= 9.88, *p*_(DFS)_= 0.015 (approx.), and *t*_(OS)_= 3.15, *df*_(OS)_= 10.31, *p*_(OS)_= 0.010 (approx.)]. The effect is further documented within, assuming the time-event associations by Kaplan–Meier analysis for DFS and OS (Figure 5).

**Figure 6:**
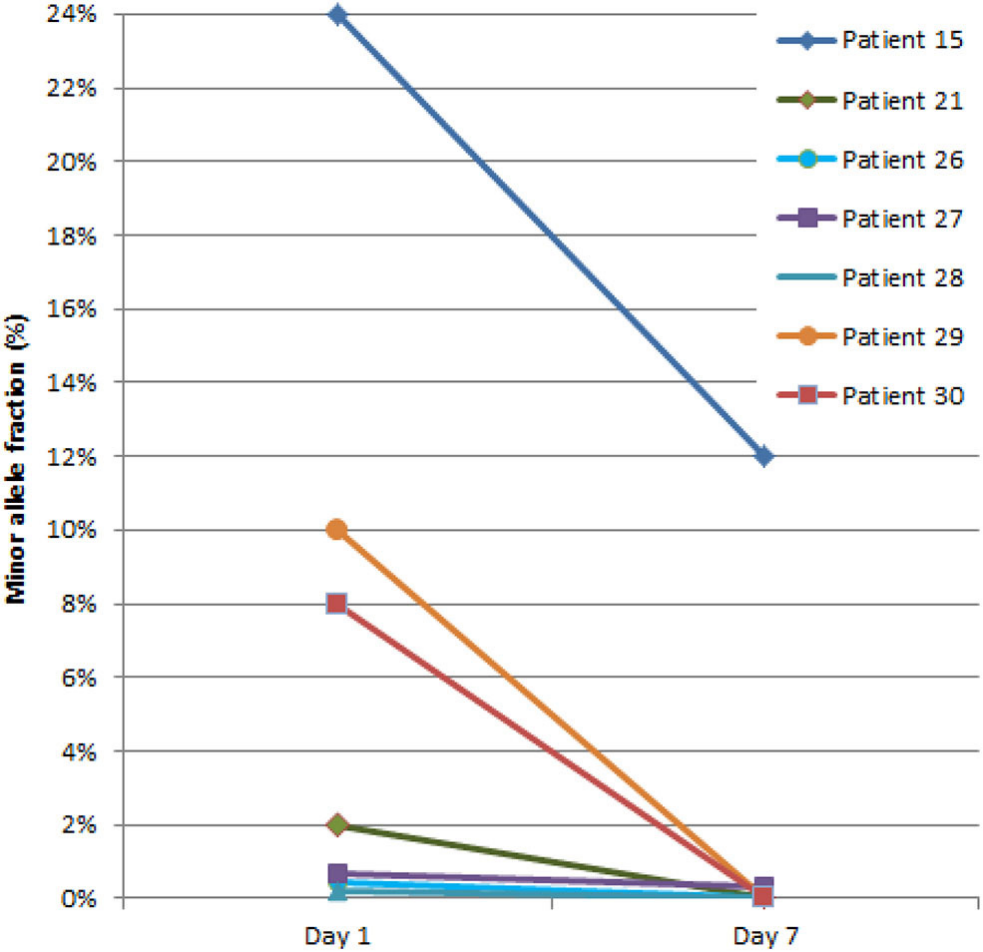
Dynamics of circulating tumor DNA (ctDNA) during the first week of neoadjuvant chemoradiotherapy (NCRT). The ctDNA quantity is presented as a percentage of DNA fragments bearing tumor-specific mutation detected in plasma [denoted as minor allele fraction (MAF)].

The early dynamics of ctDNA revealed an interesting phenomenon as shown in Figure 6. Surprisingly, during the first week of NCRT, ctDNA has been indiscriminately eliminated or significantly reduced from circulation in all patients. Accordingly, there was no association between the change in ctDNA levels (before and during NCRT) and TRG or TNM staging.

## Discussion

In this pilot study, we aimed to provide additional evidence of the effect of NCRT in LARC using various predictive biomarkers. There were several reports in the literature investigating the association between the presence of cfDNA as well as ctDNA and the prediction of an NCRT therapeutic response in rectal tumors. Zitt et al. evaluated cfDNA levels in LARC before and after NCRT and following surgical interventions (42). Studied cases were divided into NCRT non-responders and responders. The median level of pretreatment cfDNA was 4.2 ng/ml, after termination of CRT 1 ng/ml, and after surgery 4.1 ng/ml. The authors found that pretreatment levels of cfDNA of non-responders and responders do not significantly differ. At the end of treatment, cfDNA levels were higher in the non-responder cohort. In almost all patients, cfDNA levels substantially decreased toward the end of the NCRT regimen. However, the major limitation of this study is the overall small number of analyzed cases (*n* = 26).

In 2011, Agostini et al. published a set of 67 cases with LARC (43). These clinical investigators measured cfDNA levels before, during, and after NCRT. They determined the total cfDNA concentration and the proportion of long and short DNA fragments, thus establishing a “DNA integrity index.” Like Zitt et al., also, these authors did not observe a significant correlation between the pretreatment cfDNA levels and the response to NCRT. However, they provided evidence that NCRT responders had a significantly lower DNA integrity index than non-responders following NCRT.

Sun et al. have verified that cfDNA levels in CRC patients are significantly higher than in healthy subjects (44). Furthermore, they determined the plasma concentration of two DNA fragments (100 and 400 bp) before and after NCRT, and they found that the 400-bp fragment concentration was significantly lower in the responder group after termination of NCRT, thus demonstrating a higher level of fragmentation.

Carpinetti et al. performed whole-genome sequencing of tumor DNA and thus determined specific DNA fragments for each of four patients (19). The authors searched for ctDNA fragments in patients' plasma and demonstrated that in patients with good treatment response, ctDNA levels decreased during NCRT. When ctDNA increased again, it was associated with cancer progression and preceded the rise of CEA and the manifestation of recurrence detected by various imaging approaches. However, this study suffers from a rather small number of analyzed cases. Similarly, Li et al. detected ctDNA levels before and during NCRT. In this study, the prediction of treatment response based on ctDNA positivity before NCRT was 70% (45). On the contrary, Yang et al., in a larger group of patients, did not confirm the association between the pretreatment level of ctDNA and the response to NCRT in patients with RC (46).

In our study, we have hypothesized that early changes in plasma ctDNA reflect the immediate effect of NCRT and thus will be of utility in predicting its therapeutic efficiency. Hence, we expected that upon evaluation of ctDNA plasma dynamics, we will be able to differentiate NCRT non-responders, sparing them from adverse effects of continued/aggressive cancer treatment schemes. Accordingly, we have determined the ctDNA levels prior to NCRT and immediately at the end of the first week of NCRT. In addition, we aimed to find potential correlations with mutations in a panel of six commonly examined genes.

A large variation in baseline ctDNA positivity is apparent from previous reports ranging from 15 to 77% in various groups of LARC patients (47). In order to reduce high cost associated with liquid biopsy testing to enable for future cost-effective routine diagnostic approach, we have in this work employed a “cascade” approach (Figure 1). In this regard, we have always started evaluation with a relatively very simple and fast singleplex PCR method that required only a relatively small amount of samples (600 μl of plasma). This “Level 1” method, the DCE, was capable of revealing a ctDNA presence at >1% of minor allele fraction (MAF) (39, 47) in just 2 h. In case of a negative result, we performed in “Level 2” a high-resolution BEAMING assay. BEAMING (which stands for beads, emulsion, amplification, and magnetics) utilizes a digital droplet PCR resulting in amplified product of individual ctDNA fragments being bound to individual magnetic beads and subsequently detected by flow cytometry. The approach, which is performed using a dedicated instrumentation, exhibits sensitivities down to 0.01% (MAF) on residual amounts of archived ctDNA that were left after DCE Level 1 testing. In this study, only patients exhibiting tumors with *KRAS* mutation could be subjected to Level 2 testing, owing to the specificity of the BEAMING technology. Level 1 (DCE) testing revealed ctDNA positivity in five patients (5/30, 16.7%), and Level 2 (BEAMING) revealed ctDNA positivity in two more patients (2/4, 50%). The overall yield from this multilevel testing approach was 23.3% (7/30), mainly due to the limitation of BEAMING testing directed at *KRAS* ctDNA mutations only. Indeed, other high-sensitivity alternative techniques would improve this; nonetheless, the obtained frequency is comparable with previously observed results for stages II and III in rectal cancer (29).

Contrary to our expectations, we have not observed any predictive correlation between the baseline ctDNA levels and the actual outcome of NCRT in terms of TRG or TNM staging. Yet when the prognostic effect was evaluated, patients showing baseline ctDNA positivity have exhibited a shorter progression-free survival and OS (Figure 6). This is in agreement with previous work by Tie et al., who have reported ctDNA as a negative prognostic factor for the overall patient survival (48). For most ctDNA-positive patients, imaging has, indeed, subsequently revealed a presence of previously unrecognized micrometastatic sites. The ctDNA positivity should therefore be considered to guide therapy-related decisions following surgical treatment as similarly applied in breast (49) or colorectal cancer (50, 51).

Intriguingly, in all patients, we have observed a strong reduction or complete elimination of ctDNA at the end of the first NCRT week. Counterintuitively, ctDNA levels were reduced regardless of the eventual clinical outcome. Apparently, this unequivocal rapid ctDNA clearance following the therapy dose suggests presence of a more general phenomenon not related to the actual patient characteristics or specific tumor biology. The ctDNA removal from plasma is primarily a result of enzymatic digestion (34, 52). It has been reported recently that some DNA exonucleases active in DNA repair are released as a result of radiation damage (53). It can only be speculated that such a radiation-induced activity of exonucleases could result in a temporal effect of ctDNA clearance following the administration of NCRT. In order to elucidate the aforementioned phenomena, ctDNA monitoring should be performed at even shorter time intervals. Although most papers describing the use of ctDNA in palliative chemotherapy (54, 55) and, more recently, immunotherapy (56) apply monitoring with initial sampling at day 7 or later, it may well be necessary to perform examination at even shorter intervals of days or hours from the therapy start, respectively. Recently, a similar approach directed at the evaluation of ctDNA in urine has recently been recently applied for monitoring of daily dynamics of tumor response to targeted anticancer therapy in non-small-cell lung cancer (NSCLC) (57). When performed during the initial phase of the NCRT, possibly within hours from receiving the first radiation fraction together with chemotherapy and continuing for the next several days, such approach could substantiate ctDNA dynamics underlying eventual transient changes in tumor morphology and its damage, including subsequent ctDNA uptake resulting from the administered multimodal therapy. Thus, understanding of the detailed timing of ctDNA release and clearance may be essential for the long-awaited applicability of the ctDNA-based therapy outcome prediction for NCRT treatment of LARC patients (58) and beyond.

## Conclusions

In the present work, we have demonstrated the utility of monitoring of early changes in ctDNA levels in patients with LARC undergoing NCRT prior to surgical treatment. By applying a multilevel ctDNA detection approach, we were able to monitor ctDNA dynamics in seven patients receiving NCRT. We have evaluated the previously reported preoperative presence of ctDNA as a negative prognostic factor, which may be useful in direction of patients for adjuvant therapy following surgery.

We have observed a clear reduction of ctDNA levels in all patients during the initial week of NCRT, but without any direct association to the objective clinical response evaluated by TRG or TNM. As a consequence, we could not predict the response to preoperative NCRT in LARC on the basis of the ctDNA levels. Although such observations might exclude the use of early ctDNA changes as a predictive biomarker of NCRT outcome, our findings may open new research avenues on the mechanisms of ctDNA release and clearance upon cellular damage due to the combined effects of chemoradiation.

## Data Availability Statement

The raw data supporting the conclusions of this article will be made available by the authors, without undue reservation, to any qualified researcher.

## Ethics Statement

The studies involving human participants were reviewed and approved by ethics committee Motol University Hospital. The patients/participants provided their written informed consent to participate in this study. Written informed consent was obtained from the individual(s) for the publication of any potentially identifiable images or data included in this article.

## Author Contributions

FP: designed study, organized and followed up the patient cohort, collected samples, and prepared and drafted manuscript. MMi: designed study, and prepared and drafted manuscript. TH and BB: processed and analyzed samples. MMa: prepared a revised manuscript. LS: performed a statistical analysis. LB: managed molecular testing of patients and revised manuscript. JH: designed study and overseeing clinical part of project. All authors contributed to the article and approved the submitted version.

## Conflict of Interest

MMi and BB are employed by Elphogene. MMi has an ownership stake in Elphogen. LB has stock ownership in Genomac Research Institute, a private research organization. LB and TH are employed by Genomac Research Institute. The remaining authors declare that the research was conducted in the absence of any commercial or financial relationships that could be construed as a potential conflict of interest.

## Abbreviations

CEA: carcinoembryonic antigen
cfDNA: cell-free DNA
CRC: colorectal cancer
CT: computed tomography
ctDNA: circulating tumor DNA
DCE: denaturing capillary electrophoresis
DWI: diffusion-weighted imaging
LARC: locally advanced rectal cancer
MRI: magnetic resonance imaging
NCRT: neoadjuvant chemoradiotherapy
RC: rectal cancer
TNM staging: TNM Classification of Malignant Tumors
TRG: Dworak histopathological tumor regression grade.
